# Frequency-Dependent Premature Differentiation of Pheochromocytoma Cells Exhibits Band-Pass Filter Behavior Correlation with Intracellular Enzyme Activation Kinetics

**DOI:** 10.3390/ijms26115287

**Published:** 2025-05-30

**Authors:** Zubaidah Ningsih, Nguyen H. N. Tran, Andrew H. A. Clayton

**Affiliations:** 1 Department of Chemistry, Faculty of Mathematics and Natural Sciences, Brawijaya University, Jl. Veteran, Malang 65145, Indonesia; zubaidah@ub.ac.id; 2 Department of Physics and Astronomy, Optical Sciences Centre, School of Science, Computing and Emerging Technologies, Swinburne University of Technology, Melbourne, VIC 3122, Australia; 3 Department of Mechanical Engineering and Mechanics, Drexel University, Philadelphia, PA 19094, USA; nt625@drexel.edu

**Keywords:** PC12 cells, epidermal growth factor receptor, differentiation, frequency response, transfer function

## Abstract

Advances in microfluidics, optogenetics and electronics have enabled the study of dynamically controlled inputs on cellular fate. Here, we applied a microfluidic system to deliver periodic inputs of growth factors to pheochromocytoma cells and measured the extent of premature differentiation as a function of input frequency. Epidermal growth factor-triggered differentiation peaked at two cycles/hour, while nerve growth factor-triggered differentiation peaked at one cycle/hour. To interpret the results, we analyzed a published model that attributed pheochromocytoma cell differentiation to the linear combination of activated enzymes extracellular signal-regulated kinase (ERK), cAMP response element binding protein (CREB), protein kinase B (AKT) and c-Jun N-terminal kinase (JNK) at specific times after step input stimulation. Transfer functions for enzyme activation were derived from the published time-domain activation kinetics and these transfer functions were combined in a parallel architecture as a predictor of neurite outgrowth, as a function of input frequency. Qualitative agreement was observed between the model and the experiments.

## 1. Introduction

Cells, as the simplest living organisms, such as bacteria, must be able to respond appropriately to the external environment, to survive, move, grow and divide. How cells respond to the environment has been studied by two extreme yet complementary approaches. The reductionist inside-out approach focusses on which components of the cells (i.e., receptors, adaptors, enzymes and transcription factors) are activated in response to environmental cues (i.e., ligand). Connections between the activated components lead to maps of biochemical signaling pathways that are akin to wiring diagrams in electronic circuits. Cell fates are then related to which components and pathways are activated. In the complementary outside-in approach, the cell is treated as a signal processor, and one measures an integrated response, such as cell fate (or enzyme activation), as a function of changes to input frequency. The frequency response of the system gives a complete and compact picture of the system dynamics as an integrated whole (i.e., without needing to separate into components). The outside-in approach mimics biology in the sense that hormones, neurotransmitters and growth factors are not at a constant concentration in multicellular organisms, but can appear in pulses or waves. Moreover, there is a growing body of literature attesting to the role of dynamic encoding on cell fate in different signaling systems [1,2,3,4,5,6,7,8,9,10,11,12,13,14,15,16,17,18,19,20,21,22,23,24,25,26,27,28,29,30,31,32,33,34,35,36,37].

A particularly fascinating model system to study cell fate is the so-called PC12 cell system. PC12 cells are derived from rat adrenal pheochromocytoma. When treated with epidermal growth factor (EGF), PC12 cells proliferate. When PC12 cells are treated with nerve growth factors (NGF), they begin to differentiate, grow protrusions called neurites and acquire electrical properties like nerve cells. The seminal biochemical work by C.J. Marshall [1] demonstrated that transient ERK activation following EGF treatment led to cell proliferation, while sustained extracellular regulated kinase (ERK) activation following NGF treatment led to differentiation in the PC12 cells. This was one of the first experiments suggesting that cell fate could be encoded in the dynamics of an intracellular enzyme. However, EGF and NGF also trigger a multitude of pathways via their respective receptors, in addition to ERK, including cAMP Response Element-Binding Protein (CREB), protein kinase B (AKT) and c-Jun N-terminal kinase (JNK) (just to name a few). How do we reconcile these two viewpoints on cell fates and the cell environment?

To integrate the inside-out and outside-in approaches, we used a microfluidic system to deliver periodic pseudo-sinusoidal pulses of EGF or NGF to pheochromocytoma cells and measured cell differentiation as a function of input frequency (outside-in approach). The use of pseudo-sinusoidal pulses (triangular with equal on–off periods) has the advantage that the frequency response of the output can be evaluated without the complication of a frequency-dependent dose which occurs with a fixed-width rectangular pulse regime. We acknowledge that microfluidic systems have been used previously with PC12 cells to study ERK activation/cell differentiation [27] and in other cell systems [15,16,17,18,19,20,21,22,23,24,25,26]. To interrogate the inside-outside aspect of growth factor-mediated signaling in the pheochromocytoma cells, we used data from Kuroda’s lab [38] on the time-resolved activation of key signaling molecules that contribute to pheochromocytoma differentiation to predict the frequency response of individual enzyme activation (CREB, AKT, ERK and JNK) and the frequency response of differentiation. The novelty here is that a comparison of results from two different laboratories using different approaches can be placed on the same conceptual framework similar in spirit to work from the Wiley laboratory [39]. In addition, this comparison does not require a detailed signaling model of all of the components.

## 2. Results

To deliver periodic waves of growth factors to cells, we used a Y-type microfluidic device with programmable syringe pumps. The design, characterization and performance of this system have been published previously [37,40], and the system was used to demonstrate frequency-dependent population growth rates of PC12 pheochromocytoma cells in response to different frequencies of applied EGF [26]. The frequency response of PC12 cell differentiation was also examined as a function of EGF or NGF frequency [37]. In these experiments, differentiation refers to the growth of one or more neurites as thin protrusions from the main cell body. Neurites were identified using bright-field microscopy and quantified using commercial software package (Metamorph version 7.10.5) [37].

**Figure 1:**
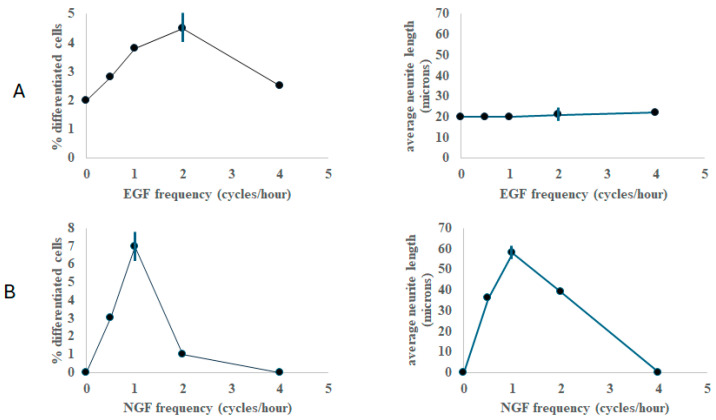
Premature differentiation (6 h) of pheochromocytoma cells as a function of growth factor frequency. *Y*-axis left panels depict the fraction of differentiated cells as a percentage. *Y*-axis right panels depict the average neurite length of the differentiated population. (**A**) Frequency response with epidermal growth factor (EGF). (**B**) Frequency response with nerve growth factor (NGF). Note the distinct maximum at two cycles/hour for EGF and one cycle/hour for NGF for the percentage differentiation. Vertical lines denote average errors, as calculated from standard deviations from measurements in triplicate across 5 frequencies. Adapted from the following source: ref. [37].

Figure 1 summarizes the results of the differentiation experiments, as a function of growth factor frequency. Note that under control conditions (constant EGF or NGF, zero cycles/hour in Figure 1) there is either undetected neurite outgrowth or a small amount of detected neurite outgrowth after 6 h of stimulation. However, with periodic stimulation, there is a clear frequency-dependent trend. For EGF, stimulation at two cycles per hour promoted the largest extent of differentiation (Figure 1A left panel). For NGF, one cycle per hour yielded the largest extent of differentiation compared to the other frequencies tested (Figure 1B left panel). For EGF, the neurite length was not strongly dependent on EGF frequency (Figure 1A right panel) at the 6-h time point. However, with NGF, frequency modulation had a measurable impact on neurite length, peaking at one cycle per hour (Figure 1B, right). It is important to stress that while the percentage of differentiated cells is very low at this early stage of observation (6 h), the apparent growth rate of the neurites in the differentiated cells is larger than under normal constant stimulation conditions (constant NGF: 0.7 microns per hour, periodic one cycle/hour NGF: 10 micron per hour). Observation of a significantly differentiated population normally takes at least 2–4 days in a continuous culture. Thus, the measure of differentiation used here is taken as a relative metric to compare different stimulus conditions at a fixed time point.

**Figure 2:**
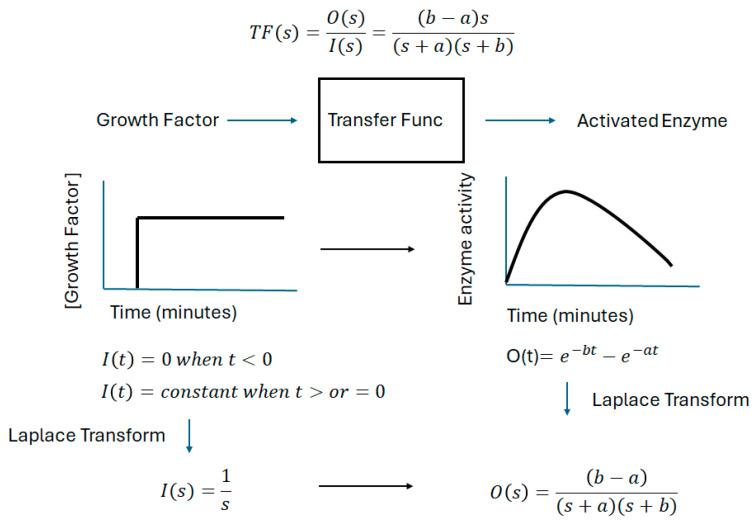
Schematic of an approach to determine the transfer function from enzyme activation kinetics following a step-function input. Fitting the time-resolved enzyme activation (phosphorylation) dynamics to the sum of two exponential functions enables the transfer function to be determined. In this figure, transfer functions have been abbreviated to either TF or Transfer Func.

As far as we are aware, a complete model for neurite outgrowth or differentiation based on the molecular interactions in a PC12 pheochromocytoma cell is not yet available. However, Kuroda’s laboratory was able to correlate different PC12 cell behaviors (proliferation, death and differentiation) with the amount of activation of intracellular enzymes ERK, CREB, JNK and AKT at certain times after stimulation [38]. Our strategy was to convert this time-domain model into Laplace space and use it to predict the extent of differentiation as a function EGF or NGF frequency. Figure 2 illustrates how knowledge of the time-dependent activation of an intracellular enzyme (following a step-function input) can be used to determine the so-called transfer function or frequency response of the system. Following the strategy of Wiley’s laboratory [39], we fit the published [38] activation transients for ERK, AKT, CREB and JNK to a sum of two exponentially decaying functions (one with positive pre-exponential amplitude, the other with a negative pre-exponential amplitude).

**Figure 3:**
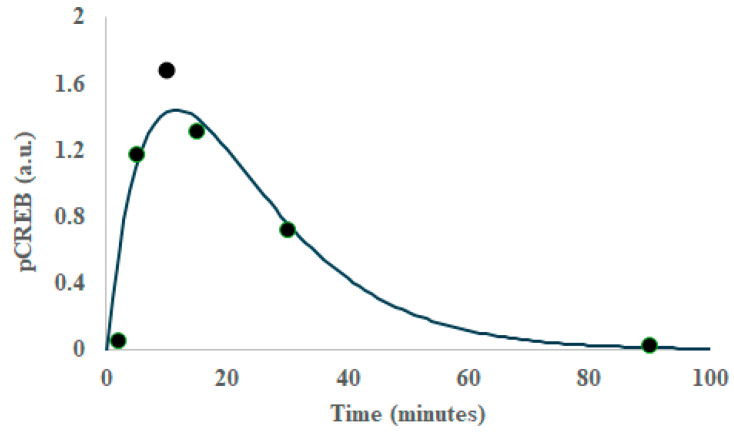
Representative plot of the activation (phosphorylation) of an intracellular enzyme (cAMP Response Element-Binding Protein (CREB) as a function of time after growth factor stimulation. The filled symbols are from experiments in reference [38] (Akimoto Y, et al. (2013) The Extraction of Simple Relationships in Growth Factor-Specific Multiple-Input and Multiple-Output Systems in Cell-Fate Decisions by Backward Elimination PLS Regression. PLoS ONE 8(9): e72780. doi:10.1371/journal.pone.0072780) and the solid line is the fit to the sum of two exponential terms. Rate parameters from the fit are collected in Table 1.

Figure 3 illustrates a typical fit to the kinetics data. Table 1 summarizes the initial conditions (EGF or NGF), the enzyme type (ERK, AKT, CREB, JNK) and the time-constants associated with the enzyme activation kinetics. Of note is that some of the enzymes had faster on–off responses than others.

**Figure 4:**
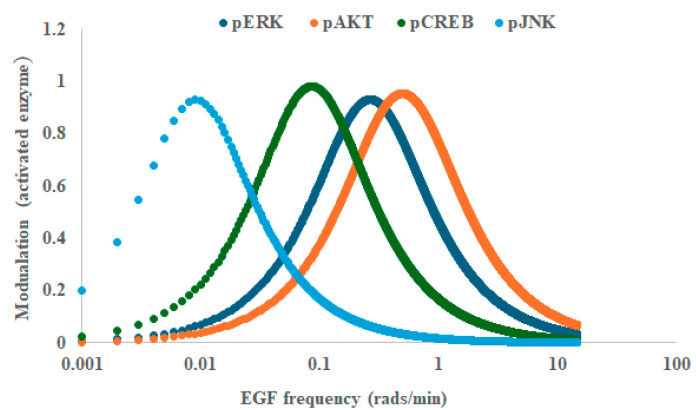
Transfer functions for epidermal growth factor EGF-triggered intracellular enzyme activation. Modulation is plotted as a function of EGF frequency. Note the distinctive frequency response of the enzymes ERK, AKT, CREB and JNK. Note the amplitudes of the curves have been manipulated so that they appear on the same scale. Protein kinase B (AKT), cAMP Response Element-Binding Protein (CREB), epidermal growth factor (EGF), extracellular regulated kinase (ERK), c-Jun N-terminal kinase (JNK).

Figure 4 depicts the calculated transfer functions derived from the enzyme activation kinetics. In Figure 4 we plotted the output modulation as a function of the input frequency. Each of the enzymes has a peak or maximum modulation at a particular frequency. That is, the molecular interaction networks leading to activation of ERK, CREB, JNK and AKT all behave as band-pass filters to growth factor concentration oscillations. It is also apparent from Figure 4 that the different enzymes have distinct frequency responses, with those positively correlated with differentiation (ERK, CREB) located in the same frequency region (i.e., within a factor of two) of around 0.1 rads/min (or one cycle/hour), where those negatively correlated with differentiation (JNK and AKT) were located at either lower frequencies or high frequencies relative to 0.1 rads/min by greater than a factor (or divisor) of (by) two.

To relate the calculated frequency responses of the different enzyme activations to the frequency response of overall cell differentiation, we noted that in Kuroda’s model [38] the cell fate response was calculated as a weighted sum of the individual responses of the enzymes at different times (i.e., neurite length = 0.95 pERK10 + 0.17 pCREB60 − 0.21 pCREB5 − 0.54 JNK30 − 0.64 pAKT5). In the transfer function parlance, this implies that the cell fate transfer function is built from the individual enzyme transfer functions in a parallel-type architecture. Simply put, because the cell outcome is a linear function of enzyme activities, we can also add transfer functions of the individual enzymes to predict cell outcome as a function of growth factor frequency.

**Figure 5:**
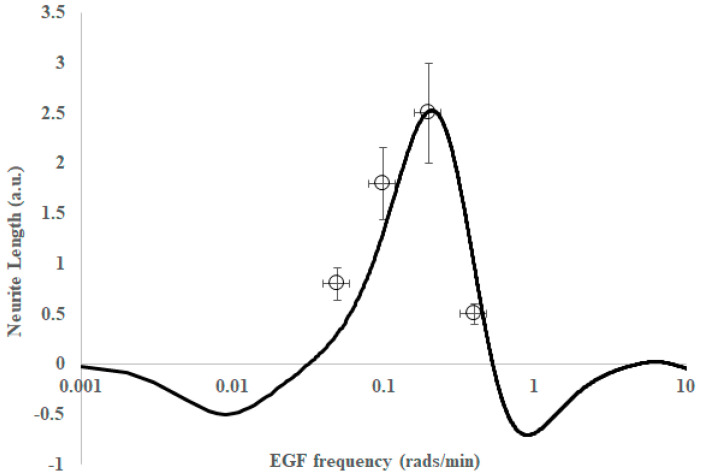
Differentiation extent as a function of epidermal growth factor (EGF) input frequency. Solid line is from the model and points are from experiments reported here. Note that the data have been normalized to allow for comparison of the frequency response only (the differentiation extent at zero frequency was subtracted from all of the data).

Figure 5 depicts the plot of the differentiation extent (a.u.) as a function of EGF input frequency (solid line is from the model, points are from experiments reported here).

**Figure 6:**
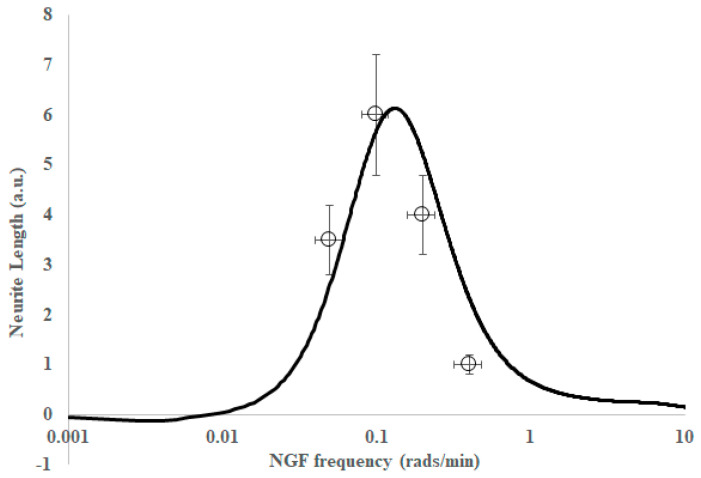
Differentiation extent as a function of nerve growth factor (NGF) input frequency. Solid line is from the model and points are from experiments reported here. Note that the data have been normalized to allow comparison of the frequency response only. The neurite length data were 36, 58, 39, 10 and divided by 10 to scale relative to the theory.

Figure 6 contains the plot of the differentiation extent (a.u.) as a function of NGF input frequency (solid line is from the model, points are from experiments reported here). Somewhat remarkably, the model appears to qualitatively account for a single positive differentiation peak (for each growth factor), a peak at a higher frequency for EGF (c/f NGF), and the fact that the peak amplitude is larger for NGF than EGF. In the next section, the discussion section, we will discuss these findings.

## 3. Discussion

Relative to constant stimulation, dynamically varying inputs appear to have an impact on cell fates. In most cell biology laboratory experiments, a constant concentration of hormone or growth factor is delivered to cells to induce them to grow, proliferate, differentiate, move or die. Recent work has revealed that the periodic delivery of hormones or growth factors in pulses, waves or sinusoids can lead to changes in cell fates relative to constant conditions [11,12,13,14,15,16,17,18,19,20,21,22,23,24,25,26,27].

Recently, Pertz’s lab [27] reported that epidermal growth factor, when delivered as fixed-width rectangular periodic pulses, was able to induce a potent differentiated state of PC12 cells at certain frequencies. This was an important discovery because, as noted above [1], the epidermal growth factor induces proliferation, not differentiation, under normal stimulation conditions (step-function). Pertz rationalized and interpreted their results using a computational/differential equation model of a receptor-activated Ras-Raf-MEK-ERK pathway with a combination of negative feedback loops [27].

In our experimental set-up, premature differentiation extent as a function of frequency resembles something called a band-pass filter. That is, there is an optimum frequency where inputs are passed to outputs. Lower or high frequency inputs are less effective at producing a differentiated state. Our results both complement and differ from those of Pertz’s lab [27], who also studied the PC12 cell system with periodic growth factor inputs. Congruent with the present results, Pertz [27] observed band-pass-like behavior for PC12 cell differentiation as a function of EGF frequency. For example, we observed the largest differentiation at a two cycles per hour input with EGF, whereas Pertz [16] observed that three pulses per hour was the most effective at promoting differentiation in PC12 cells. With NGF, however, Pertz [27] observed that higher frequency fixed-width rectangular inputs yielded greater extents of differentiation (high pass filtering), whereas we observed band-pass filtering with NGF with triangular pulse trains. We observed the largest differentiation with NGF, with a frequency of one cycle/hour, whereas Pertz [27] observed that one pulse per hour yielded the lowest differentiation (and twenty pulses/hour yielded the highest extent of differentiation). Differences in these observed effects between the two laboratories could be because the input pulses are of different shapes and on–off durations. With the fixed-width pulses of Pertz [27], the higher frequency corresponds to an increased growth factor dosage, which may account for the apparent high pass filtering behavior of PC12 cell differentiation. In contrast, for the triangular pulses used here, the average dose is invariant to changes in frequency, which may explain the band-pass filtering for both EGF and NGF. A recent theoretical frequency-domain analysis of the ERK model used in Pertz’s paper predicted differences in ERK activation responses (amplitude and shape) between rectangular pulsed inputs and triangular pulse inputs [41]. Another difference is when the differentiation was assessed (for this work it was 6 h; for Pertz [27] it was 13 h of pulsing, followed by starved medium for 24 h). More work is needed to understand the sources of the different results between the two laboratories.

To interpret our results, we have assumed that the cell responds to input-driven intracellular enzyme activation oscillations. The larger the amplitude (modulation) of the enzyme activation oscillations, the larger the overall cell response. We have used the simplest possible interpretation of enzyme activation, which comprises a single exponential rise, followed by an exponential decay and, accordingly, the simplest transfer functions for enzyme activation, which is essentially a band-pass filter. The number of data points in the enzyme activation time-series experiments limits the complexity of the model fitting function that can be employed. Similarly, in accordance with Kuroda’s model [38], we have assumed that the cell differentiation is proportional to the weighted sum of the enzyme activity of four enzymes. Because the activity modulation (and phase) of each enzyme has a unique frequency dependence, and because different enzymes are either positively or negatively correlated to differentiation, the predicted cell differentiation frequency response is also a band-pass filter.

It is important to note that the solid lines in Figure 5 and Figure 6 are not lines of the best fit to our experimental data points, but rather a prediction of the expected frequency response derived from time-domain data and a linear regression model of Kuroda [38]. As with the time-domain model, the frequency-domain model has contributions from several enzymes to the frequency response. While ERK is certainly the major contributor to differentiation, the other enzymes—CREB, AKT and JNK—contribute to a narrowing of the bandwidth of the band-pass region. The assumptions in converting the time-domain data to the frequency-domain and the use of a linear combination of transfer functions are that we operate in a linear regime, i.e., responses are independent and additive. While cells are generally non-linear, linearity can sometimes be assumed if we are operating close to a set-point.

Can the approach used here explain other results? As noted in the introduction, we reported that pheochromocytoma cells underwent EGF-frequency-dependent cell population growth rates with negative, zero and positive rates being documented [26]. According to Kuroda [38], JNK and AKT are positively correlated with cell death, while ERK and CREB are linked with cell proliferation and survival. This may partially account for the time-dependent cell population decline at low frequency (a half cycle/hour), cell population increases at one cycle/hour and the cell population decline at four cycles/hour [26]. In the low frequency triggered state, JNK is predominantly activated, while CREB and ERK are predominantly activated at one cycle/hour and AKT is predominantly activated at four cycles/hour. It is difficult to progress beyond a qualitative discussion without detailed information on what controls the actual cell population growth rates. Nevertheless, the analysis is compatible with a model wherein different input frequencies can selectively drive biochemical pathways that control cell death or cell proliferation. This requires that pathways leading to enzyme activation have distinct kinetics that are linked to a phenotypic state.

More work is needed to understand the connections between intracellular enzyme activity dynamics and cell fate. Time-lapse experiments with intracellular activity reporters on single cells and cell populations have observed correlations between cell fates and enzyme activation parameters, such as transient versus sustained signal activation, integrated signal or oscillations/pulsing [42]. Being able to control the dynamics of enzyme activation kinetics through periodic forcing may help in eliminating some of the factors thought to control cell fate. Recent work indicates that this is also a possibility with electrical inputs [43]. Finally, the contribution of mechanical effects on cell fate, i.e., shear forces of changing fluid flows, is an open question that was not addressed in this paper. Experiments under pulsed conditions (but with no growth factor) could potentially address this question, but they were not attempted in this work. We note that our experimental set-up changing the flow rate of buffer or growth factors over the cells did not induce neurite outgrowth, since cells closest to the inlets did not grow neurites. It is only where there was mixing of the two inlet solutions downstream from the inlets in the microfluidic device that optimal neurite growth was observed (i.e., where there was the greatest frequency modulation of growth factor concentration (unpublished observation).

## 4. Materials and Methods

Growth factors (EGF or NGF, Sigma-Aldrich, Castle-Hill, NSW, Australia) were delivered to cultured pheochromocytoma cells using programmable syringe pumps and microfluidic devices as described for EGF in a previous publication [26]. A constant concentration of growth factors was delivered via perfusion under gravity [26]. Prior to the experiments, cells were plated and serum was starved in a stationery condition overnight to facilitate cell attachment. The frequencies of growth factor delivery were half a cycle/hour, one cycle/hour, two cycles/hour and four cycles/hour. The amplitudes of the growth factors were 50 ng/mL of EGF and 300 ng/mL of NGF. Perfusion or periodic delivery was performed for 6 h. After 6 h, bright-field images of the cells were taken and quantified for neurites using image analysis software. Cells were counted as differentiated if they contain at least one neurite with a minimum length that is twice that of the cell body (i.e., neurite should be at least twenty microns in length).

To analyze the transient activation of ERK, CREB, AKT and JNK, the data from Kuroda’s lab publication [38] were imported into an Excel spreadsheet (Microsoft Excel, Microsoft corporation, Redmond, WA, USA). Data from the time-resolved activation kinetics for each enzyme were fit to the sum of two exponential terms, i.e.,

(1) $$I\left(t\right)=N(e^{-bt}-e^{-at})$$

In Equation (1) *I*(*t*) denotes the signal as a function of time, *t* is time (in units of minutes) and *a* and *b* are rate constants (in units of inverse minute). *N* is a normalization constant.

For the “rise and decay” kinetic model of Equation (1) (following a step input), the corresponding transfer function *T*(*s*) is given by the expression in Equation (2), where *s* is the complex frequency (s=−iω).

(2) $$T\left(s\right)=\frac{N(b-a)s}{\left(s+a)(s+b\right)}$$

To relate the transfer function to experiments carried out under periodic concentration delivery, we need the phase and the modulation as a function of the real frequency (i.e., cycle per minute). The phase θ as a function of frequency ω is given by the arctangent of the quotient of the imaginary and real parts of the transfer function,

(3) $$\theta \left(\omega \right)=ATAN\left[\frac{Im(T\left(s\right))}{Re(T\left(s\right))}\right]$$

The modulation *M* as a function of frequency ω is given by the expression,

(4) $$M\left(\omega \right)=\sqrt{{Re(T\left(s\right))}^{2}+{Im(T\left(s\right))}^{2}}$$

To calculate the cell differentiation as a function of frequency, we will need an expression for each of the activated enzyme signals at a fixed time point after the peak of the periodic input stimulation. To compute the signal, with I under periodic stimulation after a certain fixed time period and ∆t as a function of frequency ω, the expression in Equation (5) was used.

(5) $$I\left(\omega ,∆t\right)=NM\left(\omega \right)\cos(\theta \left(\omega \right)+\omega ∆t)$$

To compute the differentiation extent (neurite length, *L*) as a function of frequency (ω, in cycles per minute), the signals from the enzymes (Equation (5)) were summed according to the linear expression from Kuroda’s paper, i.e.,

(6) $$L\left(\omega ,∆t\right)=0.95{I(\omega ,10)}_{ERK}+0.17{I(\omega ,60)}_{CREB}-0.21{I\left(\omega ,5\right)}_{CREB}-0.64{I\left(\omega ,5\right)}_{AKT}$$

### Materials

PC12 cells (*Rattus novergicus* pheochromocytoma, Sigma-Aldrich, passage number 8) were the biological model used for this study. Dulbecco’s modified Eagle’s medium (Sigma-Aldrich) was prepared and supplemented with 5% l-glutamine (Sigma-Aldrich), 5% Penicillin/Streptomycin (Sigma-Aldrich), 10% equine serum (Life Technology, Carlsbad, CA, USA) and 5% fetal bovine serum (Sigma-Aldrich). Supplements were stored as aliquots at −20 °C. On the day of use, the supplements were thawed and added to the cell culture medium. The completed medium was stored at 4 °C until use.

The design, fabrication and characterization of the microfluidic chamber has been previously described [40]. The microfluidic chamber is of Y-type, with two inlet arms (each arm is 0.6 cm in length) and a cell seeding area (3.1 cm in length). The chamber is 25 µm in the vertical direction, with a total volume of 0.7 µL.

## 5. Conclusions

The cell differentiation response of pheochromocytoma cells to different frequencies of pseudo-sinusoidal modulated EGF or NGF concentrations was examined for the first time in this paper. Both EGF-triggered and NGF-triggered differentiation exhibited band-pass filter characteristics with growth-factor-distinct responses. The differentiation, as a function of frequency, was calculated from the transformed literature data on enzyme activation kinetics. A good agreement between experiments and calculations was observed. This analysis establishes a relationship between the frequency response of the cells to periodic inputs and the observed differences in enzyme activation kinetics (i.e., transient versus sustained ERK activation) from classical biochemical experiments.

## Figures and Tables

**Table 1:** Summary of extracted rate parameters a and b from the fits of transient enzyme activation kinetics to the sum of two exponential functions. Protein kinase B (AKT), cAMP Response Element-Binding Protein (CREB), epidermal growth factor (EGF), extracellular regulated kinase (ERK), c-Jun N-terminal kinase (JNK), nerve growth factor (NGF).

Growth Factor/Enzyme	A (per Minute)	B (per Minute)
EGF/JNK	0.012	0.018
EGF/CREB	0.081	0.092
EGF/ERK	0.243	0.293
EGF/AKT	0.391	0.636
NGF/JNK	0.012	0.018
NGF/CREB	0.040	0.048
NGF/ERK	0.113	0.147
NGF/AKT	0.032	0.045

## Data Availability

The original contributions presented in this study are included in the article. Further inquiries can be directed to the corresponding author(s).

## Abbreviations

The following abbreviations are used in this manuscript:

ERK	Extracellular signal-regulated kinase
JNK	c-Jun N-terminal kinase
CREB	cAMP Response Element-Binding Protein
AKT	Protein kinase B
EGF	Epidermal growth factor
NGF	Nerve growth factor
